# A New Risk Score Based on Eight Hepatocellular Carcinoma- Immune Gene Expression Can Predict the Prognosis of the Patients

**DOI:** 10.3389/fonc.2021.766072

**Published:** 2021-11-19

**Authors:** Dingde Ye, Yaping Liu, Guoqiang Li, Beicheng Sun, Jin Peng, Qingxiang Xu

**Affiliations:** ^1^ Nanjing Drum Tower Hospital, Medicine School of Southeast University, Nanjing, China; ^2^ School of Life Science and Technology, Southeast University, Nanjing, China; ^3^ Department of General Surgery, Affiliated Drum Tower Hospital, Medical School, Nanjing University, Nanjing, China

**Keywords:** hepatocellular carcinoma, immune-related genes, immune-related risk score, prognosis, nomogram

## Abstract

**Background:**

Hepatocellular carcinoma (HCC) is one of the malignant tumors with high morbidity and mortality worldwide. Immunotherapy has emerged as an increasingly important cancer treatment modality. However, the potential relationship between immune genes and HCC still needs to be explored. The purpose of this study is to construct a new prognostic risk signature to predict the prognosis of HCC patients based on the expression of immune-related genes (IRGs) and explore its potential mechanism.

**Methods:**

We analyzed the gene expression data of 332 HCC patient samples and 46 adjacent normal tissues samples (Solid Tissue Normal including cirrhotic tissue) in The Cancer Genome Atlas (TCGA) database and clinical characteristics. We analyzed the gene expression data, identified differentially expressed IRGs in HCC tissues, filtered IRGs with prognostic value to construct an IRG signature, and classified patients into high and low gene expression groups based on the expression of IRGs in their tumor tissues. We also investigated the potential molecular mechanisms of IRGs through a bioinformatics approach using Protein-Protein Interaction (PPI) network, Kyoto Encyclopedia of Genes and Genomes (KEGG) database analysis and Gene Ontology (GO) database analysis. Differentially expressed IRGs associated with significant clinical outcomes (SIRGs) were identified by univariate Cox regression analysis. An immune-related risk score model (IRRSM) was established based on Lasso Cox regression analysis and multivariate Cox regression analysis. Based on the IRRSM, the immune score of the patients was calculated, and the patients were divided into high-risk and low-risk patients according to the median score, and the differences in survival between the two groups were compared. Then, the correlation analysis between the IRRSM and clinical characteristics was performed, and the IRRSM was validated using the International Cancer Genome Consortium (ICGC) database.

**Results:**

The IRRSM was eventually constructed and confirmed to be an independent prognostic model for HCC patients. The IRRSM was shown to be positively correlated with the infiltration of four types of immune cells.

**Conclusion:**

Our results showed that some SIRGs have potential value for predicting the prognosis and clinical outcomes of HCC patients. IRGs affect the prognosis of HCC patients by regulating the tumor immune microenvironment (TIME). This study provides a new insight for immune research and treatment strategies in HCC patients.

## Introduction

Hepatocellular carcinoma (HCC), the most common primary liver cancer (accounting for over 80% of cases), is one of the malignant tumors with high morbidity and mortality worldwide. The most recent epidemiological data reveal that liver cancer ranked sixth in incidence, with 906,000 new cases, and third in cancer deaths, with 830,000 deaths, worldwide in 2020. Currently, surgical resection remains the only radical treatment for HCC. However, the surgical resection success rate is low, and the postoperative recurrence rate is high (1, 2), which severely limits the beneficial effect of surgical treatment.

HCC is the result of alterations in the interaction of a variety of cellular and molecular factors, including genetic, epigenetic, transcriptomic, and metabolic, that ultimately affect interactions in the tumor microenvironment. In particular, the tumor immune microenvironment (TIME) plays a very important role in the occurrence and treatment of HCC. The formation and development of tumors appears to be the result of the interaction between hepatocytes or HCC cells and immune cells. The immune system can recognize cancer cells and activate the immune response to eliminate tumor cells (3). Immunotherapy has become a highly promising therapeutic modality for the treatment of various cancers, including HCC (4). Concurrently, immune checkpoint inhibitors have become the most promising strategies in cancer immunotherapy (5, 6).

As a treatment modality for cancer, immunotherapy offers a promising treatment method for patients with advanced tumor. In particular, HCC is a highly heterogeneous tumor, which is closely related to immunity. Studies have revealed that some components of the TIME play key roles in the occurrence, development, invasion, metastasis and drug resistance of tumors, such as infiltrating immune cells, secreted cytokines/chemokines and other components. Research on the relationship of immunity and the development of HCC is very important (7). In the past few decades, immune-related genes (IRGs) have been shown to play a critical role in the occurrence, development and prognosis of various tumors (8, 9). IRGs can better predict the prognosis of patients and also provide a reference for early treatment of tumors. Although numerous studies have examined the correlation between IRGs and the prognosis of HCC patients, and there are differences in predictors in different studies. Therefore, additional prospective studies and basic experiments are still needed to verify the correlation between IRGs and the prognosis of HCC patients.

Accordingly, we designed this study to further investigate the clinical value of TIME and IRGs in the assessment of the prognosis of HCC patients. We obtained and identified HCC-related differentially expressed IRGs in tumor tissue samples, as well as the corresponding clinicopathological characteristics of the patients, and further evaluated the relationship between IRGs and overall survival (OS). We undertook to develop an IRGs-based immune-related risk score model (IRRSM) to predict the prognosis of HCC patients. This study revealed the likely mechanism of the role of IRGs in the progression of HCC, and established a suitable and accurate model providing a fresh perspective for clinical decision-making.

## Methods

### Data Downloading and Preprocessing

Total RNA‐sequencing data of 368 HCC tissue samples and 50 adjacent normal tissues samples were obtained from The Cancer Genome Atlas (TCGA) database (https://cancergenome.nih.gov/). After excluding patients with a shorter survival time (the patients with OS ≤90 days were excluded as these patients probably died of some unpredictable factors, such as severe infections, hemorrhage and liver failure), 332 hepatocellular carcinoma tissues and 46 adjacent tissues were eventually included. RNA expression sequencing data and patient clinical information of 237 HCC samples were also obtained from the International Cancer Genome Consortium (ICGC) database (https://dcc.icgc.org/) for validation.

### The Analysis of Differential Genes and Differential IRGs

The limma package in the R software (https://bioconductor.Org/packages/release/bioc/html/limma.html) was used to screen differentially expressed genes (DEGs) between HCC and adjacent non-tumor tissues. The threshold used to screen for DEGs was set as follows: “False Discovery Rate (FDR) < 0.05 and log2| FC |> 1”. We obtained 192 IRGs from the ImmPort database (https://immport.niaid.nih.gov), and through the intersection with the identified DEGs, a total of 102 IRGs were ultimately obtained. In order to investigate the interaction between these genes, we mapped the Protein-Protein Interaction (PPI) network of these immune genes using the STRING online database. The CytoHubba in Cytoscape software version 3.8.2 is used to display the PPI network results. Additionally, we perform gene enrichment analysis using the Gene Ontology (GO) and Kyoto Encyclopedia of Genes and Genomes (KEGG) databases to investigate the potential molecular mechanism of the roles of these IRGs. The GO and KEGG pathways were identified using the cluster profiler, org.Hs.eg.db and enrichplot packages in the R software.

### Construction of the IRG Signature

We performed a single-factor Cox regression analysis of the IRGs to determine the relationship between the expression of IRGs and the OS of HCC patients. In order to avoid overfitting and delete highly related genes, Lasso Cox regression was performed using the survival and glmnet package in the R software. Genes detected using the Lasso algorithm were evaluated by stepwise multivariate Cox regression analysis. Risk scores were obtained based on genes expression multiplied by a linear combination of the regression coefficient obtained by the multivariate Cox regression analysis. Patients were categorized into high-risk and low-risk groups according to the median risk score. The Kaplan–Meier analysis was performed to compare OS between high-risk and low-risk groups using the survival package in R.

We verified the model internally and externally, and subsequently tested the feasibility of the model through the clinical decision-making curve. The CIBERSORT (http://cibersortx.stanford.edu/) database was used to assess the levels of tumor infiltrating immune cells, which contains immune-related scores for various genes. Therefore, we scored according to the number of IRGs expressed in each sample, and eventually obtained the immune score of each sample. We determined the correlation between IRRSM and immune cell infiltration.

In order to confirm the relationship between IRGs associated with significant clinical outcomes (SIRGs) and the clinicopathological characteristics of HCC patients, we analyzed the correlation between the IRRSM and clinicopathological characteristics. The Tumor, Nodes and Metastasis (TNM) staging system is a well-recognized and the most commonly used system for classifying the extent of tumor cancers. Cancer stage defined according to the TNM classification system takes into account overall tumor burden and metastatic extent. Likewise, microvascular invasion and histopathological grading are closely associated with patient prognosis and treatment response. In order to explore the potential molecular mechanism SIRGs, we construct a regulatory network between SIRGs and transcription factors (TFs) based on NetworkAnalyst 3.0 (www.networkanalyst.ca).

### Statistical Analysis

In order to predict the median survival and 1-, 3-, 5-year survival probability of HCC patients, we used the rms package in the R software to construct a nomogram. To verify the independent predictive ability of the nomogram model, the receiver operating characteristic (ROC) curve was drawn using the survival ROC package in the R software, and the models of different single clinical indicators and nomogram were used to evaluate the prognostic predictive ability of patients. We also showed the degree of calibration of the model through the calibration curve. Univariate Cox regression analysis, Lasso regression analysis and multivariate regression analysis were used to confirm SIRGs. The Kaplan-Meier curve was used to estimate the OS of the high-risk and low-risk groups and verify the independent prognostic factors of HCC patients. A radargram was drawn by the fmsb package in the R software, which displays the relationship of the IRRSM and level of immune cell infiltration. All statistical analyses were performed using SPSS26.0 software (IBM Corporation, Armonk, USA). P < 0.05 is considered statistically significant. All experiments were performed 3-4 times to reduce the error.

## Results

### Differential Gene Expression Analysis

**Figure 1:**
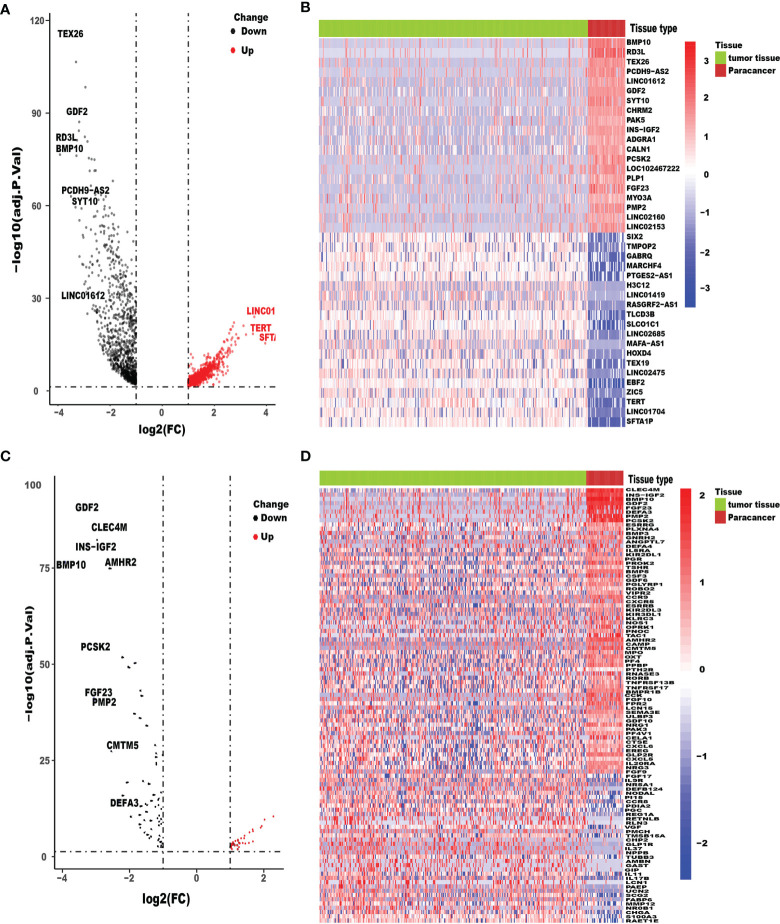
Differentially expressed HCC genes and immune-related genes. Volcano plot **(A)** and heatmap **(B)** illustrated the differentially expressed genes between HCC tissues and para-carcinoma tissues. Immune-related genes (IRGs) with different expression levels were showed in the volcano plot **(C)** and heatmap **(D)**. FDR < 0.05 and log_2_FC |> 1.

We acquired the RNA-seq gene expression data and clinicopathological data of a total of 418 HCC patients with tumor tissues or adjacent normal tissues from TCGA database. In addition, after excluding patients with incomplete clinicopathological data and survival time of less than 90 days (36 HCC tissue samples and 4 adjacent normal tissues), patients-related data were extracted for the remaining 378 samples. Ultimately, the expression data of 332 HCC tissue samples and 46 adjacent normal tissues and their respective clinicopathological characteristics were included in this study. After conversion of 34,428 Ensembl human gene ID into gene names, 2,024 DEGs between HCC tissues and adjacent normal tissues, with threshold set as: (log2| FC |>1 and FDR <0.05), were identified using the limma package, of which 961 were downregulated and 1,063 were upregulated (**Figure 1A**). The top 20 upregulated and downregulated mRNAs of DEGs were visualized by heatmaps as shown in **Figure 1B**. We found 102 IRGs, of which 67 were downregulated and 35 were upregulated genes (**Figure 1C**). The expression level of IRGs is illustrated by a heatmap (**Figure 1D**).

### The Characteristics of IRGs

**Figure 2:**
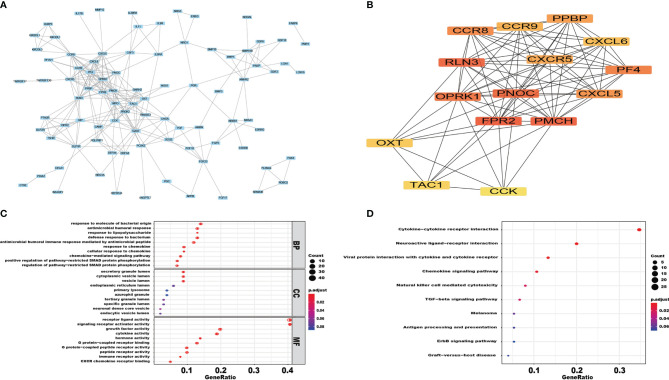
The functional enrichment analysis of differentially expressed IRGs. PPI network **(A)** of IRGs and the hub IRGs **(B)**. The top pathways of IRGs were shown in biological process (BP), cellular component (CC), molecular function (MF) **(C)**, and top KEGG pathways **(D)**.

To investigate the mutual interaction among IRGs, a PPI network was constructed, as shown in **Figure 2A**. In this PPI network, FPR2, PMCH, RLN3, PNOC, OPRK1, PF4, CCR8, CXCL5, PPBP, CXCR5 were regarded as the hub genes among the 102 IRGs (**Figure 2B**). We performed GO term, KEGG pathway, and functional enrichment analyses to explore the potential biological functions of the common immune-related DEGs. As shown in the **Figure 2C**, “response to molecule of bacterial origin”, “secretory granule lumen” and “receptor ligand activity” were the most enriched terms in the biological processes (BP), cellular components (CC) and molecular functions (MF) categories, respectively. “Cytokine−cytokine receptor interaction” was identified to be the most enriched among the KEGG pathways of the IRGs (**Figure 2D**).

### Clinical Outcomes of the High-Risk and the Low-Risk Groups

**Figure 3:**
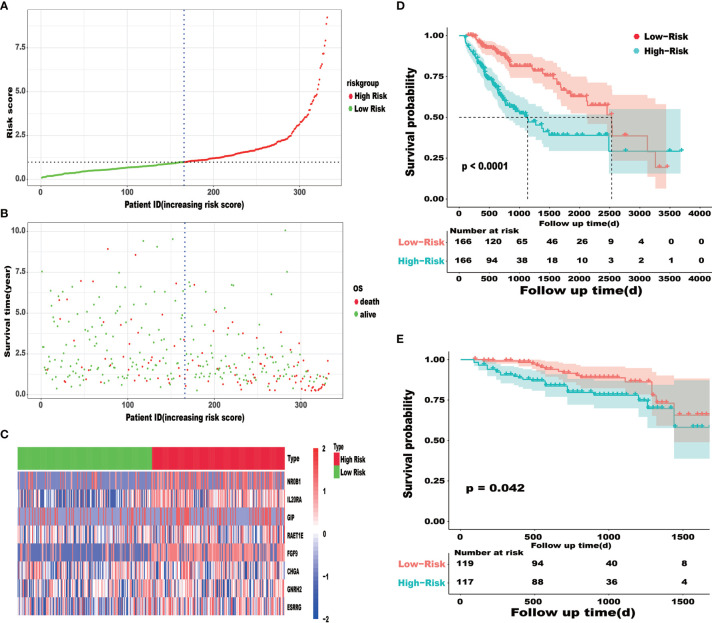
IRRSM was created by SIRGs **(A–C)**. The distribution of high-risk group and low-risk group **(A)**. Survival status of the high-risk and the low-risk group **(B)**. The heatmap of the expression levels of twelve SIRGs **(C)**; Survival curve for the high risk and low risk groups of HCC patients in the TCGA database **(D)**. Survival curve for the high risk and low risk groups of HCC patients for validation in ICGC database **(E)**.

In order to establish the IRRSM, we identified 8 statistically significant SIRGs (including six high-risk IRGs and two low-risk IRGs) among the 21 SIRGs of the univariate Cox regression analysis (**Table 1**) using Lasso regression analysis and multivariate Cox regression analysis (**Table 2**). According to the IRRSM, the HCC patients were divided into the high-risk group and the low-risk group (**Figure 3A**). The mortality of patients with the higher risk scores was significantly higher than that of patients with the lower risk scores (**Figure 3B**). There were distinct differences in the expression levels of the 8 genes between the high-risk and low-risk groups (**Figure 3C**). In addition, in the IRRSM, the survival probability of the low-risk group was significantly higher than that of the high-risk group (**Figure 3D**). To further verify the predictive capacity of the IRG signature, 236 HCC patients from the ICGC database were used for external validation. To this end, we divided the patients into high-risk and low-risk groups. Consistent with the result obtained with TCGA dataset, the K‐M analysis showed that patients in the high‐risk group had a worse prognosis than those in the low‐risk group (**Figure 3E**).

**Table 1:** The results of univariate Cox regression.

gene	HR	HR .95% Low	HR .95% High	*p-Value*
FABP6	1.06	1.03	1.10	<0.001
NR0B1	1.06	1.03	1.09	<0.001
FGF9	1.07	1.03	1.11	0.002
CHGA	1.07	1.02	1.12	0.003
CXCL5	1.05	1.02	1.09	0.003
GAST	1.04	1.01	1.07	0.004
PAEP	1.04	1.01	1.07	0.004
MMP12	1.06	1.01	1.10	0.008
GIP	1.04	1.01	1.08	0.010
IL20RA	1.05	1.01	1.08	0.012
GNRH2	0.96	0.93	0.99	0.015
CXCL6	1.04	1.01	1.07	0.016
RAET1E	1.10	1.01	1.19	0.023
LCN1	1.04	1.00	1.08	0.031
UCN2	1.04	1.00	1.08	0.033
CTSE	1.04	1.00	1.07	0.036
ESRRG	0.95	0.91	1.00	0.036
IL17B	1.04	1.00	1.09	0.037
SCG2	1.05	1.00	1.09	0.043
AMBN	1.04	1.00	1.07	0.047
PLXNA4	0.95	0.90	1.00	0.049

HR, Hazard ratio.

**Table 2:** The results of multivariate Cox regression analysis.

gene	HR	HR .95% Low	HR .95% High	*p-Value*
IL20RA	1.043	1.004	1.083	0.029
GIP	1.038	1.002	1.074	0.036
NR0B1	1.047	1.015	1.08	0.003
RAET1E	1.114	1.028	1.207	0.008
FGF9	1.064	1.02	1.11	0.004
CHGA	1.064	1.017	1.113	0.007
ESRRG	0.093	0.884	0.979	0.006
GNRH2	0.962	0.931	0.995	0.024

HR, Hazard ratio.

### Development and Validation of Prognostic Signature

**Figure 4:**
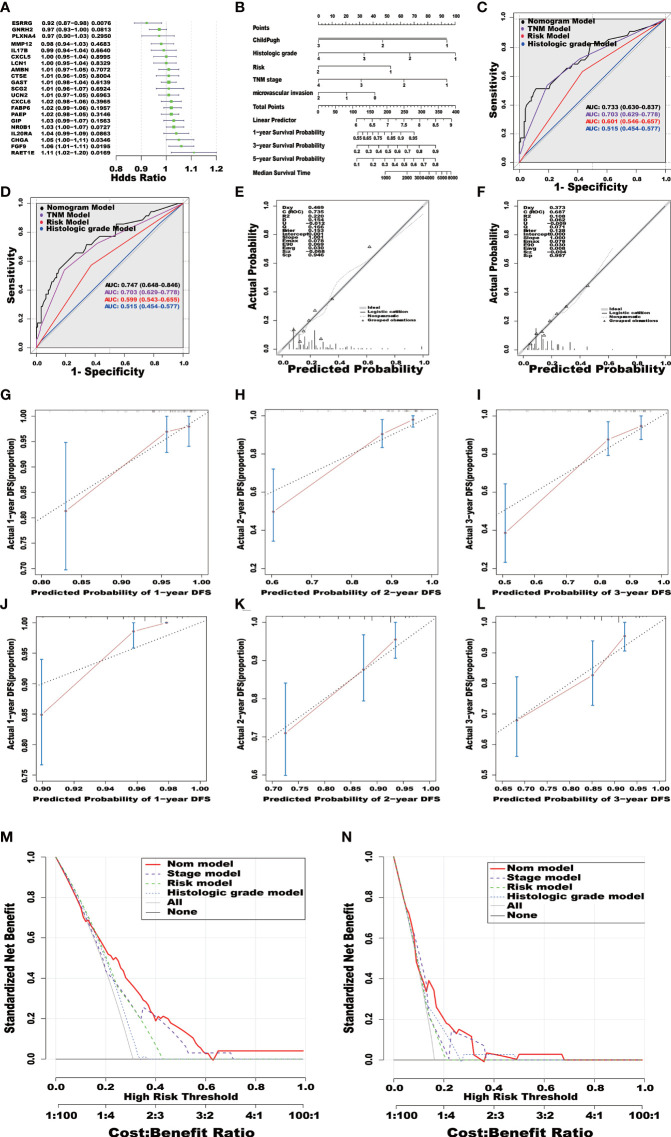
Independent prognostic prediction analysis **(A)**. The clinical features, including Child Pugh class, histologic grade, TMN stage and microvascular invasion, were combined with Risk Score to construct the nomogram model **(B)**. The survival predicted ROC curves of four distinct models, namely, Nomogram model, TNM stage model, Risk score model and histologic grade model were compared in TCGA and ICGC database **(C, D)**. The calibration curve diagrams of the training set **(E)** and the validation set **(F)** have good agreement between the predicted probability and the actual probability, both S: p>0.05. The calibration curves of 1,2,3-year survival **(G–L)**. The dotted line represented the ideal prediction model, and the red solid line represented the observed model in TCGA database **(G–I)**. The calibration curves of 1,2,3-year survival. The dotted line represented the ideal prediction model, and the red solid line represented the observed model in ICGC database **(J–lL)**. Decision Curve Analysis model: A clinical decision-making model based on the final multiple risk factors in TCGA and ICGC database **(M, N)**.

The Child Pugh score, histologic grade, TMN stage (tumor, metastasis, node), Risk score and microvascular invasion degree were included in TCGA cohort. Histologic grade, TMN stage and Risk score were included in ICGC cohort. A total of 332 patients in TCGA cohort and 236 patients in the ICGC cohort with complete clinical information were included. The hazard ratio (HR) was calculated and expressed in a forest plot (**Figure 4A**). According to the multivariate independent prognostic analysis results of TCGA cohort, a prognostic nomogram including significant factors (P-value < 0.05) was developed and constructed with the rms package in the R software (**Figure 4B**). Receiver operating characteristic (ROC) curves of the nomogram model, risk score model, histologic grade model, and TMN stage model were plotted using survival ROC package in the R software (**Figures 4C, D**). The area under the ROC curve (AUC) was also provided. Additionally, we compared the calibration and the discrimination inside and outside the model (**Figures 4E, F**). The calibration curves were also plotted and revealed the good prognostic prediction efficacy of the model (**Figures 4G–L**). The assessment of the Clinical Impact of Risk Prediction Models with Decision Curves is shown in **Figures 4M, N**.

### Relationship of the IRG Signature and Clinical Variables

**Figure 5:**
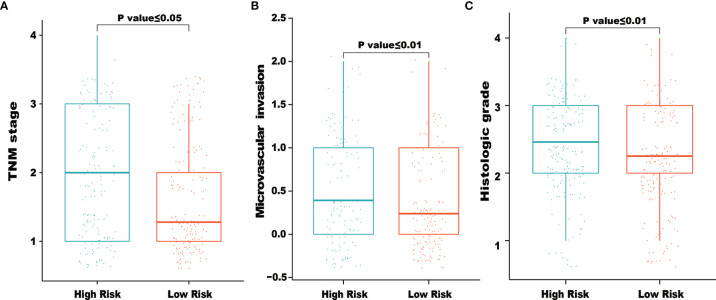
Relationships between risk score and clinical characteristics. The figures demonstrated that higher of TNM stage **(A)**, microvascular invasion **(B)** and histologic grade **(C)** tended to correlated with higher risk scores.

We also examined the correlations between clinical factors and risk scores calculated from the IRG signature. As shown in **Figure 5**, the results indicated that higher TNM stage, microvascular invasion and histologic grade tended to correlate with higher risk scores. The correlations between the eight IRGs and clinical tumor histologic grade were also assessed. As shown in **Supplementary Figure 1**, higher expression of NR0B1 and IL20RA were associated with higher tumor histologic grade (**Supplementary Figures 1E, F**), and lower expression of ESRRG were associated with higher tumor histologic grade (**Supplementary Figure 1G**).

### The Analysis of the Immune Status of the High-Risk and Low-Risk Groups

**Figure 6:**
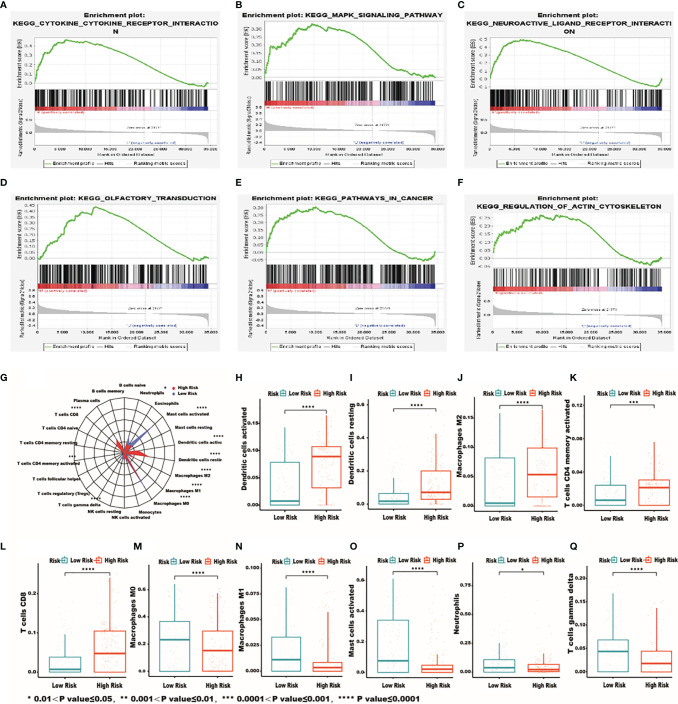
The most significantly enriched KEGG pathways between high- and low-risk patients. KEGG: cytokine-cytokine receptor interaction **(A)**; MAPK signaling pathway **(B)**; neuroactive ligand receptor interaction **(C)**; olfactory transduction **(D)**; pathway in cancer **(E)**; regulation of actin cytoskeleton **(F)**. Relationships between the IRRSM and immune cells. The relationships between IRRSM and immune cells infiltration: radar plot **(G)**; dendritic cells activated **(H)**; dendritic cells resting **(I)**; macrophages M0 **(J)**; T cells CD8 **(K)**; B cells naive **(L)**; macrophages M1 **(M)**; macrophages M2 **(N)**; mast cells activated **(O)**; mast cells resting **(P)**; T cells gamma delta **(Q)**.

The functional enrichment analysis of the IRG signature was performed by gene set enrichment analysis (GSEA) to compare the high‐risk and the low‐risk groups in HCC. The KEGG pathway enrichment analysis revealed that “cytokine-cytokine receptor interaction”, “MAPK signaling pathway”, “neuroactive ligand receptor interaction”, “olfactory transduction”, “pathway in cancer”, “regulation of actin cytoskeleton” were significantly related to the high‐risk group (**Figures 6A–F**). In order to further examine whether the IRRSM accurately reflects the TIME, we analyzed the relationships between the IRRSM and immune cells infiltration (**Figure 6G**). We found that activated dendritic cells (**Figure 6H**), resting dendritic cells (**Figure 6I**), non-activated (M0) macrophages (**Figure 6J**) and CD8 T cells (**Figure 6K**) showed positive correlation with risk score, whereas naïve B cells (**Figure 6L**), classically activated (M1) macrophages (**Figure 6M**), alternatively activated (M2) macrophages (**Figure 6N**), activated mast cells (**Figure 6O**), resting mast cells (**Figure 6P**) and gamma delta T cells (**Figure 6Q**) showed the opposite results. These results motivated us to further pursue the research on the underlying functions and mechanisms in future studies.

### Mechanisms of the Immune‐Related Signature

**Figure 7:**
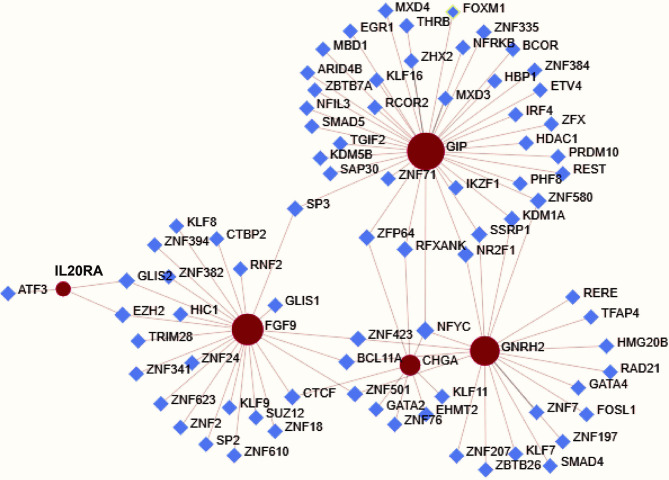
Mechanisms of the immune‐related risk signature. Transcription factors‐based regulatory network with the eight immune‐related genes in the signature.

We explored the potential regulatory mechanisms of the eight immune‐related genes that made up the signature, which could reflect the regulatory mechanisms of the signature. Based eight immune‐related genes, we constructed a relevant regulatory network between TFs and immune‐related genes by NetworkAnalyst 3.0 (www.networkanalyst.ca) (**Figure 7**). In terms of the figure, we can see that GIP, GNRH2, FGF9, CHGA and IL20RA were included in the regulatory network, while there was no regulatory relationship between the RAET1E, NR0B1, ESRRG and TFs.

## Discussion

With the rapid development of next-generation sequencing technologies, our understanding of transcriptional alterations in HCC has greatly improved. The molecular landscape of synchronous HCC reveals genetic heterogeneity and predicts the prognosis of patients, thereby providing an innovative and effective way to identify promising markers and targets for HCC diagnosis and treatment (10–14). According to the survival prediction of patients with prognostic biomarkers, individualized treatment can be better developed in clinical practice (15, 16). Compared with a single gene, a multigene-based model has been shown to be more robust and precise in diagnosis and prognosis in many cancers (17, 18). Thus, it provides new options for prognostic prediction and treatment of patients.

Since generally people have an in depth understanding of the importance of immune activity in tumorigenesis, progression and prognosis, the emerging cancer immunotherapy approaches have always had an attention-catching position in the field of cancer treatment (19). Over the past few decades, numerous targeted cancer and immunotherapeutic drugs have been and are currently being developed, with many already used in routine clinical care, including pembrolizumab, ipilimumab and nivolumab, which have been approved for clinical application and have achieved good results (20, 21). Therefore, the discovery of more promising and sensitive immune-related biomarkers, such as IRGs is necessary to improve the efficacy of immunotherapy. In this study, we developed a novel prediction method based on IRGs in HCC patients, which may also independently predict the OS and median survival of HCC patients. Most importantly, we also investigated the molecular mechanisms related with the IRG signature using the bioinformatics tools and analysis of the correlations between our IRG signature and immune cell infiltrations to evaluate immune cells infiltration. These findings indicate that some clinical signature is of great significance for predicting the prognosis of HCC patients and exploring potential immunotherapy targets in HCC.

In this study, a novel IRG signature was identified to classify HCC patients according to the risk score. We integrated multiple genes into a single signature through multivariate Cox regression analysis modeling. The novel IRG signature was verified to be a significant predictor both in TCGA and ICGC cohorts. The eight genes included in the identified IRG signature were CHGA, RAET1E, FGF9, GIP, NR0B1, IL20RA, ESRRG and GNRH2. The heatmaps were plotted to illustrate the risk of the identified genes based on gene expression profile. As shown in the heatmaps, the expression of the twelve altered genes increased together with the risk score in both cohorts.

The signature was constructed by eight IRGs that were able to predict prognosis of HCC patients. In this study, all eight TRGs were shown to be independent prognostic markers for HCC patients.

Expression of CHGA has been reported to be associated with prognosis in colorectal cancer (22–24), and is generally recognized as the main biomarker for neuroendocrine neoplasms (25, 26). Recently, CHGA has been proposed as an early diagnosis biomarker for gastric cancer (27), prostate cancer (28), and pancreatic neuroendocrine tumors (29). However, there is no study about CHGA expression and early diagnosis in HCC. ESRRG has been identified as a tumor suppressor gene in several cancers (30), which is consistent with our findings that ESRRG is lowly expressed in HCC tissue and can predict the prognosis of HCC patients, indicating that ESRRG could regulate the occurrence and development of HCC. FGF9 was first identified from the secretions of human glioma MCF-G1 cells, and found to promote NIH-3T3 cell line malignant transformation, suggesting that FGF9 may be a tumor-promoting factor (31). Indeed, FGF9 has been found to be highly expressed in HCC, and significantly associated with the proliferation of cancer cells (32–34). Several alterations in FGF-signaling have been found to affect liver carcinogenesis (35). Aberrant expression of the endocrine FGF19 and its high affinity FGFR4 receptor contributes to HCC progression (36). GIPR is found in many cancers, including the majority of pancreatic, ileal, and bronchial neuroendocrine tumors (NETs). The high GIPR content in a broad spectrum of cancers, makes GIPR a potential target for cancer diagnostic and therapeutic purposes (37, 38). Expression of the gonadotropin-releasing hormone homologue peptides GNRH2 and their receptor GNRHR has been demonstrated in various malignancies. Studies have shown that GNRH2 is closely related to the occurrence of multiple cancers, and to affect the prognosis of patients. Recent studies have provided evidence that IL20RA signaling regulates the development of certain cancers. Another study found that IL20RA expression was elevated in breast cancer and colorectal cancer (39). Epigenetic modification of NR0B1 leads to its ectopic activation in Ewing’s sarcoma and lung cancer, enabling it to promote cancer cell proliferation (40). Additionally, it can also inhibit the proliferation of liver cancer cells by suppressing the transcriptional activity of β-catenin (41). It has been reported that RAET1E might impair NKG2D-mediated NK cell cytotoxicity to liver cancer cells (42).

This study established a signature for calculating the risk score based on the expression levels of eight genes, which predicted the median survival and OS of HCC patients. Moreover, this study also provides an innovative analysis approach to identify significant biomarkers. Also, through comprehensive data analysis, reliable results with excellent repeatability can be obtained, which can be verified with external data and clinical outcomes.

## Conclusion

In this paper, we analyzed the roles of SIRGs in the prediction and evaluation of the clinical prognosis of HCC patients and verified the predictive value of some SIRGs. We establish a reliable and accurate IRRSM to predict the prognosis of HCC patients, which offer a new perspective for treatments by early intervention.

## Data Availability Statement

The datasets presented in this study can be found in online repositories. The names of the repository/repositories and accession number(s) can be found in the article/**Supplementary Material**.

## Author Contributions

DY, JP, and QX designed the study. DY and YL analyzed the data and wrote the manuscript. JP and QX provided technical expertise and support. The manuscript was prepared and reviewed by DY, YL, GL, and BS. All authors contributed to the article and approved the submitted version.

## Conflict of Interest

The authors declare that the research was conducted in the absence of any commercial or financial relationships that could be construed as a potential conflict of interest.

## Publisher’s Note

All claims expressed in this article are solely those of the authors and do not necessarily represent those of their affiliated organizations, or those of the publisher, the editors and the reviewers. Any product that may be evaluated in this article, or claim that may be made by its manufacturer, is not guaranteed or endorsed by the publisher.

## Supplementary Material

Supplementary Figure 1 Relationships between clinical tumor histologic grade with expression of the eight immune‐related genes. CHGA **(A)**, RAET1E **(B)**, FGF9**(C)**, GIP **(D)**, NR0B1 **(E)**, IL20RA **(F)**, ESRRG **(G)**, GNRH2 **(H)**.
