# Supplementary figures and images for: A New Risk Score Based on Eight Hepatocellular Carcinoma- Immune Gene Expression Can Predict the Prognosis of the Patients
Source: Front Oncol. 2021 Nov 19;11:766072. doi: 10.3389/fonc.2021.766072 (PMC8639602; doi:10.3389/fonc.2021.766072)

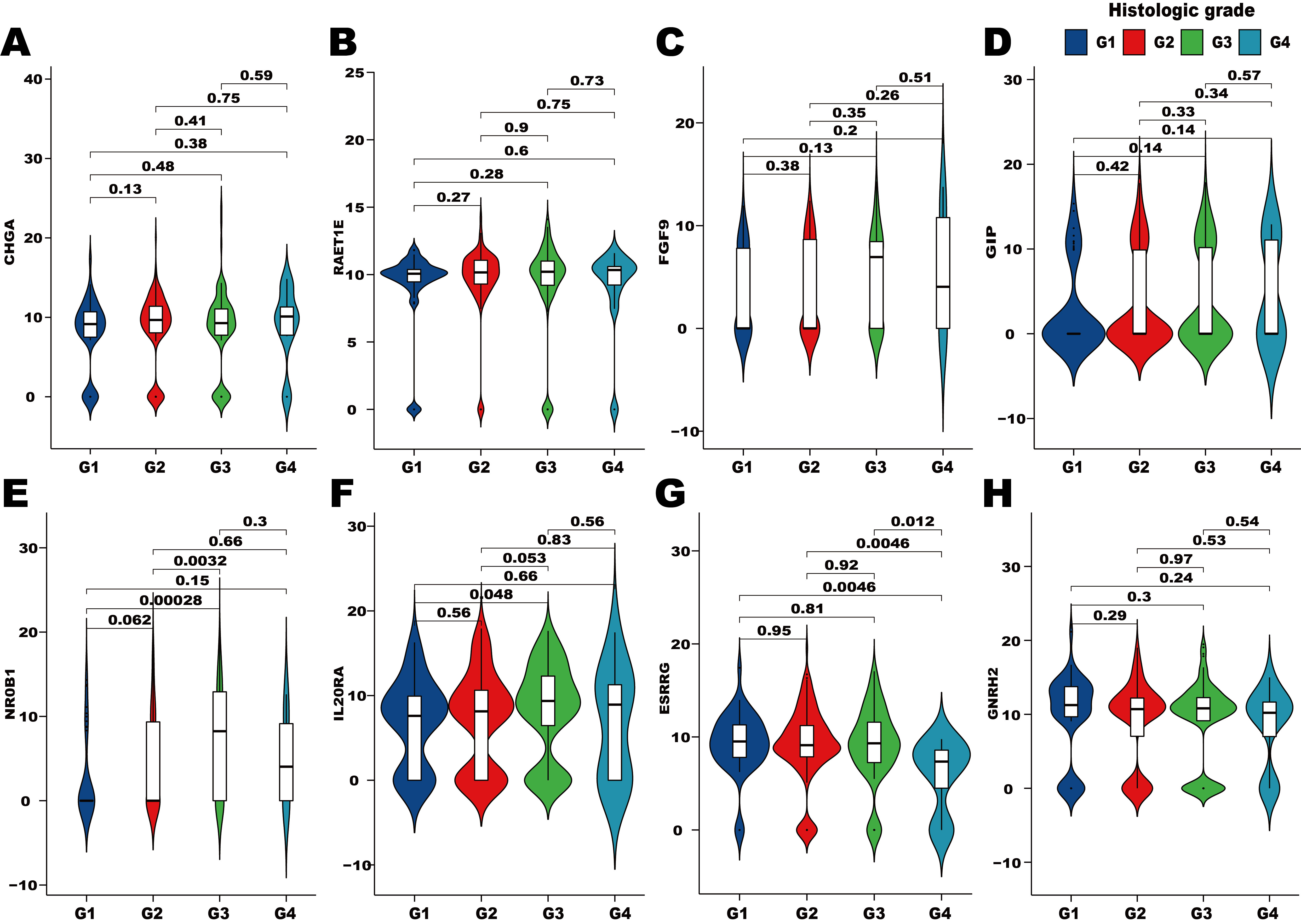

Supplement: Supplementary Figure 1 — Relationships between clinical tumor histologic grade with expression of the eight immune‐related genes. CHGA (A), RAET1E (B), FGF9(C), GIP (D), NR0B1 (E), IL20RA (F), ESRRG (G), GNRH2 (H). [file Image_1.jpeg]
